# 1726. Characterization of humoral immune responses to COVID-19 vaccination in children in Canada: interim analysis from the Severe acute respiratory syndrome-related coronavirus 2 PRevalence In children and youNG adults in British Columbia (SPRING) Study

**DOI:** 10.1093/ofid/ofad500.1558

**Published:** 2023-11-27

**Authors:** Gabrielle N Gaultier, Mandy Lo, Brynn McMillan, Amy Lee, Bing Cai, Tony Harn, Sofia R Bartlett, Muhammad Morshed, Inna Sekirov, Kate Zinszer, Elinor Simons, Piushkumar Mandhane, Jonathon L Maguire, Agatha N Jassem, Manish Sadarangani

**Affiliations:** University of British Columbia, Vancouver, BC, Canada; University of British Columbia, Vancouver, BC, Canada; University of British Columbia, Vancouver, BC, Canada; University of British Columbia, Vancouver, BC, Canada; University of British Columbia, Vancouver, BC, Canada; University of British Columbia, Vancouver, BC, Canada; BC Centre for Disease Control, Vancouver, British Columbia, Canada; University of British Columbia, Vancouver, BC, Canada; University of British Columbia, Vancouver, BC, Canada; University of Montreal, Montreal, Quebec, Canada; University of Manitoba, Winnipeg, Manitoba, Canada; University of Alberta, Edmonton, Alberta, Canada; St. Michael’s Hospital, Unity Health Toronto, Toronto, Ontario, Canada; University of British Columbia, Vancouver, BC, Canada; University of British Columbia, Vancouver, BC, Canada

## Abstract

**Background:**

A vaccine series consisting of two doses of coronavirus disease 2019 (COVID-19) mRNA vaccines plus one booster dose is currently recommended for healthy children aged 5-11 years in Canada. Additional studies are needed to understand the longitudinal immunity to COVID-19 vaccination in the pediatric population. The Severe acute respiratory syndrome-related coronavirus 2 PRevalence In children and youNG adults in British Columbia (SPRING) sub-study aimed to specifically investigate antibody responses to COVID-19 vaccination in children aged 5-11 years.

**Methods:**

Fifty-four children without immunocompromising conditions or taking immunosuppressive medications were enrolled prior to dose one or dose two of their COVID-19 vaccine. Responses were quantified via: anti-index virus spike protein specific IgG (S-IgG) geometric mean concentrations (GMCs) (binding antibody units, BAU/mL) and S-IgG avidity reported as total relative IgG avidity index (TRAI) (avidity units, AU) at pre-dose two, as well as one and six months post-dose two. A Welch’s t-test compared responses between timepoints.

**Results:**

The cohort consisted of 55% male and 45% female with a median age of 8 years. The interval between first and second doses ranged from 7 – 18 (median 11) weeks. Concentrations of S-IgG increased from pre-dose two to one month post-dose two (222 vs. 1152 BAU/mL, p < 0.0001). GMCs then decreased by six months post-dose two (1152 vs. 343 BAU/mL, p < 0.0001) to levels similar to pre-dose two (p = 0.0696). TRAI increased between time points. By six months post-dose two, S-IgG TRAI (68 AU) remained higher than pre-dose two (46 AU, p < 0.0001) and one month post-dose two (65 AU, p = 0.0495). This increase in avidity was due to higher concentrations of medium high, high and very high avidity S-IgG as well as a decrease in very low avidity S-IgG at six months post-dose two.

**Conclusion:**

Concentrations of S-IgG waned significantly by six months post-dose two. The results suggest that due to repeated exposure to the S-protein, affinity maturation has resulted in the production of higher avidity antibodies post-dose two. This suggests that both S-IgG concentrations and avidity should be considered when determining protection against COVID-19 and need to be further explored to support future booster recommendations.

**Disclosures:**

**Sofia R. Bartlett, PhD**, Abbvie: Advisor/Consultant|Abbvie: Grant/Research Support|Cepheid: Advisor/Consultant|Gilead: Advisor/Consultant|Gilead: Grant/Research Support **Manish Sadarangani, BM BCh, FRCPC, DPhil**, GlaxoSmithKline: Grant/Research Support|Merck: Grant/Research Support|Moderna: Grant/Research Support|Pfizer: Grant/Research Support|Sanofi Pasteur: Grant/Research Support|Seqirus: Grant/Research Support|Symvivo: Grant/Research Support|VBI Vaccines: Grant/Research Support

